# Potential Role of AGR2 for Mammalian Skin Wound Healing

**DOI:** 10.3390/ijms24097895

**Published:** 2023-04-26

**Authors:** Anastasiya V. Kosykh, Maria B. Tereshina, Nadya G. Gurskaya

**Affiliations:** 1 Center for Precision Genome Editing and Genetic Technologies for Biomedicine, Pirogov Russian National Research Medical University, 117997 Moscow, Russia; avkosyh@gmail.com (A.V.K.); ma-treshka@yandex.ru (M.B.T.); 2 Shemyakin-Ovchinnikov Institute of Bioorganic Chemistry, 117997 Moscow, Russia

**Keywords:** skin repair, regeneration, mammals, wound healing, anamniotes regeneration, Anterior Gradient proteins, proteins disulfide isomerase

## Abstract

The limited ability of mammals to regenerate has garnered significant attention, particularly in regard to skin wound healing (WH), which is a critical step for regeneration. In human adults, skin WH results in the formation of scars following injury or trauma, regardless of severity. This differs significantly from the scarless WH observed in the fetal skin of mammals or anamniotes. This review investigates the role of molecular players involved in scarless WH, which are lost or repressed in adult mammalian WH systems. Specifically, we analyze the physiological role of Anterior Gradient (AGR) family proteins at different stages of the WH regulatory network. AGR is activated in the regeneration of lower vertebrates at the stage of wound closure and, accordingly, is important for WH. Mammalian AGR2 is expressed during scarless WH in embryonic skin, while in adults, the activity of this gene is normally inhibited and is observed only in the mucous epithelium of the digestive tract, which is capable of full regeneration. The combination of AGR2 unique potencies in postnatal mammals makes it possible to consider it as a promising candidate for enhancing WH processes.

## 1. Introduction

The skin, with its vast surface area, is the body’s largest organ and serves a multitude of functions. Among these, its protective role is paramount: the skin is the organ most frequently subjected to damage over the course of a lifetime. However, it is evident that there has been a decrease in regenerative potential throughout the evolution of vertebrates. Cold-blooded vertebrates, such as fishes and amphibians, are the champions of scarless wound healing and are capable of regenerating intricate structures such as tails, limbs, heart or eyes after accidental loss. For example, the axolotl (*Ambystoma mexicanum*) can heal deep skin injuries within 90 days, resulting in the complete replacement of damaged tissue without scarring. This newly regenerated tissue is fully functional and indistinguishable from non-injured tissue [1]. The ability of *Xenopus laevis* froglets to heal skin damage without scarring is age dependent. Post-metamorphosis froglets demonstrate scarless skin regeneration; but by 15 months of age, *Xenopus laevis* frogs start to form scar-like tissue upon wounding [2,3]. In mammals, scarless wound healing only occurs during early embryogenesis. This phenomenon was first observed in 1979 and subsequently confirmed in many studies [4,5,6]. The process of scarless wound healing (WH) has been observed in fetuses of mice, rats, pigs, monkeys and humans, with regenerative potential depending on the stage of development of the species [7,8,9]. The ability for scarless WH disappears after about 24 weeks of development in humans and at the E18.5 stage in mice (mouse pregnancy averages 20–21 days) [10,11]. In addition, it is worth noting that the larger the area of damage, the more difficult it is for the body to regenerate, which means that the ability to close a scratch without forming a scar lasts longer than the ability to heal a full-thickness wound [8]. In adult mammals, wound healing is incomplete and results in the formation of scars and a reduced number of appendages (follicles and glands).

The analysis of the molecular basis of skin injury healing in *Xenopus* froglets and axolotl has shown the important roles of the NO-generating enzyme iNOS, signaling molecules TGFβ-1 and 2, transcriptional factor prx1 and suppressor of cytokine signaling SOCS3 [12]. Importantly, some of these molecules act for a shorter time and with less intensity than in scar-forming wound healing in mammals. Additionally, both epimorphic regeneration and skin wound healing are regulated by transcriptional factor prx1, which is activated in the initial stages of *Xenopus* epimorphic limb regeneration [13]. Examination of 2.4-kb *prx1* limb-specific enhancer activity showed that it is strongly activated in mononuclear cells under the wound epidermis in *Xenopus* froglets but not in mouse skin [2]. Nevertheless, the activity of prx1 in mice is crucial for skeletal stem cell populations found within the bone marrow, periosteum and muscles that contribute to bone regeneration [14].

Yokoyama and authors suggested that induction of this *prx1* enhancer activity may be a reliable marker for therapeutically induced scarless WH in mammals [2]. Further exploration of the potential pathways for regulating prx1 expression in mammalian WH is of great interest in the context of seeking methods to modulate skin regeneration. In general, using accumulated knowledge of amphibian molecular regulators of wound healing and regeneration could possibly help to develop ways to stimulate scarless wound healing in mammalian skin. One possible approach worth investigating is the use of homologs of amphibian secreted proteins that could potentially influence the healing process without necessitating significant genetic modifications to mammalian cells. Anterior Gradient proteins (AGR) are evolutionary conserved factors, which play an important role in limb regeneration in anamniotes, may still be active in mammalian embryo development and could serve as effective regulators. In this review, we will focus on the AGR proteins, describing their structure, function as regulators of amphibian regeneration and potential role at different stages of the mammalian skin wound healing process. 

## 2. AGR Family of Protein Disulfide Isomerases (PDI) 

### 2.1. AGR Protein Structural Characteristics

AGR proteins belong to the large evolutionary conserved family of proteins with disulfide isomerase activity (PDI) [15]. Initially found and characterized in the early development of *Xenopus laevis*, AGR genes were shown to be involved in the specification of dorsoanterior ectodermal fate, i.e., in the formation of the cement gland and induction of the forebrain fate of *Xenopus* [16,17]. According to the refined phylogeny, proposed in 2013, there are three subfamilies: AG1, AGR2 and AGR3 [18]. The AG1 protein subfamily is specific for anamniotes, while AGR2 and AGR3 are present in all classes of vertebrates.

**Figure 1:**
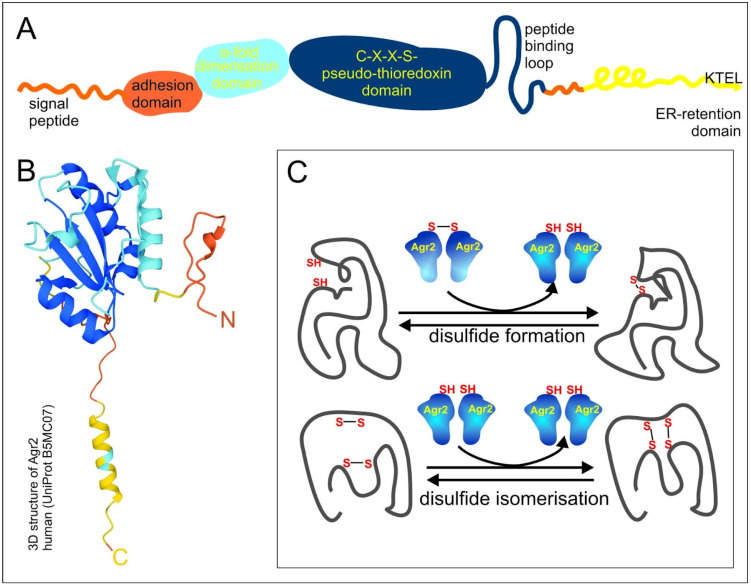
Scheme of AGR domain organization and functioning. (**A**) The scheme of AGR2 domain organization. Orange, blue, dark blue and yellow colors designate N-end, dimerization domain, pseudo-thioredoxin domain and peptide binding loop, and endoplasmic reticulum (ER)-retention domain, respectively. (**B**) The 3D structure of human AGR2 according to the UniProt Database (UniProt B5MC07). The colors of the domains are the same as in 1A. (**C**) The scheme of AGR2 functioning. Proteins with disulfide isomerase (PDI) activity catalyze disulfide formation and disulfide isomerization of client proteins.

Three PDI-like proteins: The AGR1, AGR2 and AGR3 proteins share a common domain organization, which comprises the N-terminal domain, including the signal peptide followed by a fragment of amino acids (a.a.), 21–40, which determine cell adhesion rate, and the α1 helix, a.a. 58–67, responsible for dimerization due to electrostatic interactions (the numbers of a.a. correspond to AGR2) (Figure 1A). The central pseudo-thioredoxin-domain (CXXS) is surrounded by hydrophobic a.a., which are suggested to play a role in target protein binding - peptide binding loop. The last important feature is the C-end endoplasmic reticulum (ER)-retention domain KTEL in AGR2, EDEL in AGR1 and QSEL in AGR3, which provide reverse recycling of proteins when they enter the secretory pathway and are highly important for AGR functional activity (Figure 1A,B) [19,20].

### 2.2. AGR Proteins in Anamniotes Regeneration

**Figure 2:**
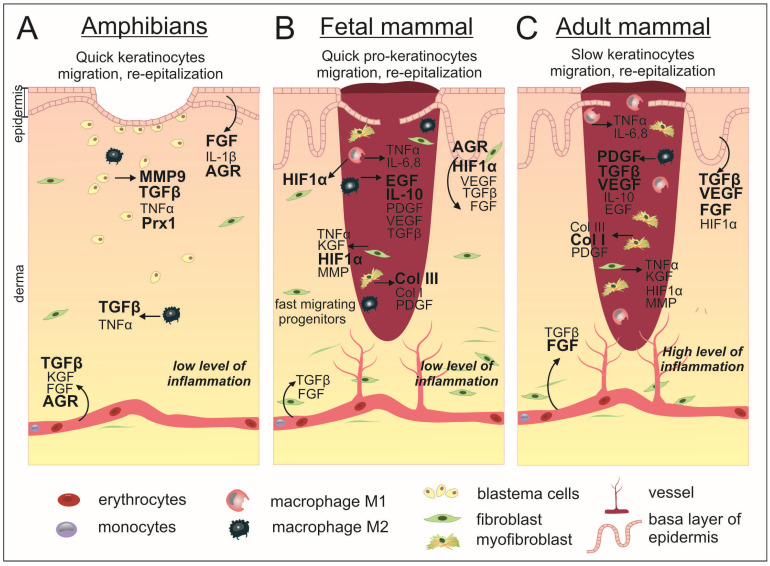
Scarless wound healing occurs in anamniotes (**A**) and mammalian embryos (**B**) as opposed to incomplete regeneration in adult mammals (**C**). In both classes of animals, at the first moment after injury, the process of coagulation starts and the bleeding stops. The resulting clot in mammals completely fills the wound, while in anamniotes the clot is much smaller and the wound surface is closed due to rapid re-epithelialization taking place on the first days after injury. The mammalian embryo WH is also characterized by accelerated migration of cellular progenitors, which leads to a shorter wound closure time compared to the postnatal period. In this connection, subsequent inflammatory processes are reduced in comparison with adult mammals. In addition, the embryo showed a smaller number of macrophages and a reduced proportion of pro-inflammatory M1. In anamniotes, the dermis is restored through the formation of a blastema by dedifferentiated cells in the wound area, while in mammals, fibroblasts proliferate and migrate to achieve dermal restoration. WH in anamniotes is regulated by specified factors whose expression level increases after injury. During embryonic development, intact skin is characterized by the presence of HIF-1α, while after injury, the expression of AGR2 is found in the epidermis. Additionally, there is an increased expression level of EGF and anti-inflammatory IL-10, as well as a higher percentage of collagen III in comparison to postnatal wounded skin. Adult mammalian wounds are characterized also by a high level of PDGF, TGF-β, VEGF, and FGF expression and the predominant production of collagen I, which forms the scar tissue. The factors with stronger activation are shown by bold.

Anamniotes are capable of complete regeneration of complicated structures such as hind or forelimbs, tails, hearts, and eye retinas. The first step of regeneration involves the formation of the wound epidermis, followed by the second and extremely important period of blastema formation underneath the wound epidermis. The blastema consists of de-differentiated, actively proliferating cells. Regeneration is the ultimate form of wound healing. Decades of investigation into regeneration on cellular and molecular levels have resulted in a detailed description of the cellular mechanisms and molecular patterns of a spectrum of key regulators involved in tissue recovery. As it turns out, anamniotes regeneration and mammalian wound healing have many common points, beginning from cellular movements to the spectrum of regulatory molecules orchestrating these processes (Figure 2) [21]. In the process of studying wound healing in amphibians, a weak immune response after injury was shown [1]. The EGF pathway and the expression of MMP9 are activated after damage in the blastema area [3,17]. In experiments with *Xenopus laevis*, a high level of expression of FGF8, 10 in the late blastema was shown [13]. In addition, in old *Xenopus*, an increase in the proportion of XTGF-β2 expression was noted in comparison with the anti-scarring XTGF-β3, which is actively expressed in younger frogs [3]. The disparities in regeneration between anamniotes and mammals lie in the timing of certain signaling processes and the participation of specific factors in anamniotes’ regeneration, which are produced by wound epithelium and induce blastema formation underneath it. The interest in such factors has led to a more detailed study of Agr genes during anamniotes regeneration. The activity of Agr genes was investigated in several anamniotic models: fish *Danio rerio*, newt *Notophthalmus viridescens* and frog *Xenopus laevis*. In all these species activation of AGRs expression was detected at the early stages of fin/limb/tail regeneration. The newt homolog of AGR2, nAG, is expressed firstly in the nerve sheath and afterwards in some glandular structures in the wound epithelium, and its expression is able to induce Cos7 cell proliferation [22]. During *Danio rerio* fins regeneration, activation of Ag1 expression is observed mainly in wound epithelium and in 1day post-amputation blastema. Further, its downregulation leads to a decrease in regeneration effectiveness [23]. In *Xenopus laevis* tadpoles, expression of Ag1 and Agr2 was detected in the wound epithelium of the regenerating tail and hindlimb at 1–2 days post-amputation. Importantly, in contrast to evolutionary conserved Agr2 and Agr3, Ag1 genes are present only in cold-blooded vertebrates; and in *Xenopus laevis* tadpoles, they are also expressed in blastema cells. As warm-blooded vertebrates lack the ability to form blastema and, respectively, cannot regenerate by epimorphosis, the authors hypothesize that the loss of the Ag1 gene could be among the prerequisites for this decrease in regeneration. Indeed, if Agr genes were blocked, the tadpole’s tail regeneration was inhibited accompanied by mitotic activity and signaling pathway inhibition. Additionally, in contrast, if these genes were overexpressed in a temporary regeneration incompetent stage (refractory period), regeneration reactivation was observed [17,18]. These data point to the importance of AGR secretory PDI for regeneration stimulation and appear to be promising objects for a detailed study of their role in the skin of amniotes in normal conditions and during wound healing.

### 2.3. Ambivalent Mammalian AGR Proteins

Mouse Anterior Gradient-2 (mAGR2) protein is the orthologous of *Xenopus laevis* cement gland protein AGR2. Briefly, the importance of AGR2 factors in WH is based on the observations of their active role during WH both in vitro and in vivo [24]. Here, we regard the structure of AGR2, its expression pattern and its functions to highlight the specifics of its activity in relation to regeneration.

Murine Agr2 expression was originally described in mRNA screening as a differentially expressed gene in the intestinal goblet cells, which line the intestines and secrete mucin [25]. The expression of mAGR2 was indiscernible in the skin tissue of newborn or adult mice, while the skin of 14-day embryos showed positive AGR2 expression [24]. Mice with a knockout mutation in the mAgr2 gene (mouse Anterior Gradient-2 gene) were viable but displayed high susceptibility to colitis. Investigation of the AGR2 null mouse phenotype and epithelial cell lineages of the gastrointestinal tract of these mice revealed that AGR2 supports the balance between differentiated and stem epithelial cells of the glandular stomach [19]. These mice exhibit premature death due to intestinal obstruction. Loss of AGR2 induced the decline of terminally differentiated cells in gastric mucous neck and intestine in favor of progenitor and stem cells with high expression of SOX9. These data indicate that AGR2 and SOX9 coordinate co-expression affects the fate of immature progenitors of epithelial cells.

Human homologs of mAgr (AGR2 or hAG2 and AGR3) are found indispensable for mucin secretion, their expression is localized in tissues, enriched with epithelial cells. AGR2 deficiency induces mucus barrier dysfunction and infantile inflammatory bowel disease in humans [26]. Later, AGR2 was shown to associate with Reptin, which belongs to the AAA+ superfamily, whose members can exist in equilibrium between monomeric apo forms and ligand bound hexamers [27]. Among the specific AGR2 client proteins is the oncogenic receptor EpCAM, which was found by affinity chromatography with an artificial peptide followed by a Proximity Ligation Assay and by in vivo colocalization fluorescence microscopy with AGR2 and EpCAM mutants [28]. AGR1, AGR2 and AGR3 were found in exocytic vesicles of cells being secreted.

According to the human protein and tissue atlas, AGR2 is expressed at a significant level in the gastrointestinal tract, respiratory tract and reproductive system (https://www.proteinatlas.org/ENSG00000106541-AGR2/tissue, accessed on 18 November 2021). AGR2 mRNA and protein are present at high levels in several metastatic adenocarcinomas [29]. AGR2 was found to be enriched in various solid human tumors: ovary, breast, gastric and lung adenocarcinomas [30,31,32]. AGR2 is secreted as it was found in patients’ blood samples. Therefore, it may be a useful biomarker for cancer diagnostics and prognosis [28]. AGR3 and AGR2 genes were found downregulated in Basal-Like Breast Cancer (BLBC) of ER-luminal breast cell lines (ER+, PR+ or - and HER2+ or -) and upregulated in non-BLBC cell lines, the latter was shown to have less recurrence and less metastatic progression [33]. High levels of both AGR2 and MUC4 were shown in patients in more differentiated regions of pancreatic ductal adenocarcinomas, where elevated protein levels were associated with shorter patient survival [34].

Apparently, each separate function of the AGR2 protein must find correlation with its multidomain structure (Figure 1).

The thioredoxin-like domain is responsible for PDI activity. It acts similar to a molecular chaperon in the ER, e.g., by participating in the folding of cysteine-rich transmembrane receptors and enabling their trafficking. It catalyzes the formation of disulfide bonds and their shuffling (isomerization) (Figure 1C). The physical interaction of AGR2 with epithelial growth factor receptor (EGFR) within the ER was shown, followed by its trafficking to the Golgi apparatus and the plasma membrane [35]. AGR2 participates in molecular chaperon machinery and interacts specifically with different peptides. It was shown that AGR2 associates with BiP/GRP78, a central molecule that regulates ER processes and initiates the unfolded protein response (UPR) signaling pathway [36]. The authors have shown that in a human colon adenocarcinoma cell line homo-dimerization of AGR2 occurs through intramolecular disulfide bond formation, and the dimeric form of AGR2 is capable of activating UPR signaling. Overexpression of the wild-type AGR2 protein induces phosphorylation of the key UPR kinases IRE-1α and PERK. Being phosphorylated, these molecular transducers dissociate from BiP/GRP78 and transmit the ER stress axis signal downstream. It was reported that ER stress-induced cell death is decreased in AGR2-overexpressed cells. Prolonged chronic ER stress induces the overexpression of AGR2 in a normal Human Pancreatic Duct Epithelial cell line [37]. A mouse model was used to study the role of mAGR2 in the etiology.

There is also an alternative way of homodimerization of the AGR2 protein by covalent S-S bond formation between C81 residues from thioredoxin-like domains, in particular under hydrogen peroxide oxidation [38]. Mutation of C81S disrupts completely homo-dimerization of AGR2 and also blocks intracellular interaction with the amino terminal MUC2 [26]. The experiments with deletion-mutations revealed that the domain with residues 21–40 is responsible for the migration activity of AGR, whereas the EALYK motif from a.a. 60–64 stabilizes its homodimeric state. The mutant with deletion of N-terminal 40 a.a. residues had a more stable dimeric state than full size protein. The monomer–homodimer equilibrium of AGR2 (with a constant of dissociation (Kd) of 8.83 μM) shows that dimerization is not so indispensable for its activity [20]. Mutant AGR2 E60A with an abolished dimeric form was shown to keep its adhesion function, which means the monomeric form of AGR2 is sufficient to provide this function. Indeed, endogenous AGR2 exists in dimeric and monomeric forms. Tight control of AGR2 dimerization prevents cells from the activation of pro-inflammatory cascades through either secretion of AGR2 or autophagy-dependent processes [39].

Why do the proteins of the AGR family with PDI activity, which participates in the regulation of protein quality control systems in the ER, act in wound healing, influencing the migration of keratinocytes and fibroblasts? Being mainly anchored in ER, the protein presumably has different functions connected with different protein–protein interactions in normal and abnormal conditions. The dual functions of AGR proteins include the important pro-oncogenic role connected with extracellular localization of the proteins and the independent ER-localized foldase function and secretory roles.

AGR2 protein exists in an equilibrium between homodimeric and monomeric states, with the first state dominating in physiologically normal conditions and the balance shifting to the monomer in cancer-associated conditions. The increased expression of endogenous mAGR2 was shown during wound healing of mouse full-thickness skin excisions, especially in the migrating epidermal tip [24]. The AGR2 protein supports cell adhesion in normal conditions (for example, in the accumulation of dorsoanterior ectoderm during development), but the same molecule is used as a pro-oncogenic signal in the development of different adenocarcinomas. Stable expression of AGR2 in the NIH3T3 cell line led to cellular transformation and induced tumor formation in nude mice after xenotransplantation of NIH3T3 AGR2+ cells [40]. Both forms of intracellular and extracellular AGR2 bind with estrogen receptor α (ER-α) and enhance IGF-1 signaling. Human AGR2 and AGR3 expression are considered as prognostic markers for hormone-responsive breast tumors [41].

The role of AGR2 in cell proteostasis was investigated by engineering a panel of cell lines with stable expression of AGR2 wild-type in AGR2 null cells. SILAC analysis was applied to trace the reprogramming changes in cells after the launch of AGR2 expression [42]. Remodeling of the cellular proteome due to AGR2 expression was conducted via suppression of the p53 signaling pathway. The expression of tumor susceptibility gene 101 (TSG101) as a cell cycle regulator was induced by AGR2 superexpression.

WH in pancreatic cancer cell lines PANC-1 with different forms of AGR2 revealed the enhanced activity of secreted AGR2 (AGR2 without the KTEL motif) in comparison with the cells with the wild-type AGR2 and control PANC-1 cells. PANC-1 cells with overexpression of AGR2-△KTEL demonstrated increased migratory and invasion capacities [28]. Elevated AGR2 expression levels were shown in seven of nine pancreatic cancer cell lines [43]. AGR2 was shown to be present in the extracellular space, serum, and urine of patients with cancer [44].

The AGR2 client proteins exhibit diverse localization patterns across various cellular compartments. Specifically, Reptin is found in the cytoplasm, RIP140 in the nucleus, calnexin (CANX), BiP (PRP78), HSP70, ERD21-23 and KDEL receptors in the ER, TMED2 in the Golgi, and EGFR and EpCAM on the cell surface plasma membrane. Additionally, AGR2 stabilizes HIF1-α and interacts with VEGFP and FGF2. The eAGR2 protein being secreted binds to ECM component laminin and regulates epithelial morphogenesis and tumorigenesis [27,36,39,44,45]. The murine model that employed caerulein-induced pancreatitis demonstrated the AGR2-induced EGFR signaling, which is necessary for tissue regeneration and the outcome of pancreatitis [46].

In this review, we analyze the physiological role of the AGR proteins from the side of their participation at different stages of the regulatory network involved in WH. In order to identify potential interactions between AGR and regulatory cascades of wound healing, we disassembled the wound healing process step by step, paying special attention to those factors, whose interactions were shown in various models in vivo and in vitro.

## 3. Mammalian Acute Wound Healing: Stages and Molecular Regulators

Wound healing is a complex and coordinated cascade of events that can be subdivided into four overlapping phases: hemostasis, inflammation, proliferation, and remodeling. This process is controlled by the interaction of numerous factors, which can simultaneously affect several phases at once.

In the first phase vasoconstriction, platelet aggregation, degranulation and blood clot formation occur. Then, the recruitment and differentiation of macrophages take place in the inflammatory stage. Next steps are re-epithelialization, angiogenesis, collagen synthesis and ECM formation, which occur during the proliferation stage. Finally, during the last stage, in addition to collagen remodeling, the final formation of the vasculature takes place. Below, we briefly concern the main processes of WH from the point of view of those key molecular events, which have possible AGR influence.

### 3.1. Hemostasis

The coagulation process initiates immediately after injury. The early regeneration processes are activated by growth factors, cytokines released from damaged blood vessels and degranulated platelets. Destruction of blood vessels results in the formation of a blood clot composed of fibrin, fibrinogen and fibronectin with accumulated platelets. A blood clot normally forms in the first few hours after injury, stops blood loss, prevents infection penetration and makes a matrix for cell accumulation and a reservoir of growth factors needed in the later stages of the healing process [47,48]. Blood clot platelets secrete PDGF, which was shown to be chemotactic for cells recruited in WH processes such as monocytes and fibroblasts. Further, PDGF increases fibroblast proliferation and correspondingly ECM production. Furthermore, PDGF promotes fibroblasts to assume the functional characteristics of myofibroblasts [49]. During the initial stages of skin wound healing, both TGF-β and TNF-α are actively secreted (Table 1) [50].

**Figure 3:**
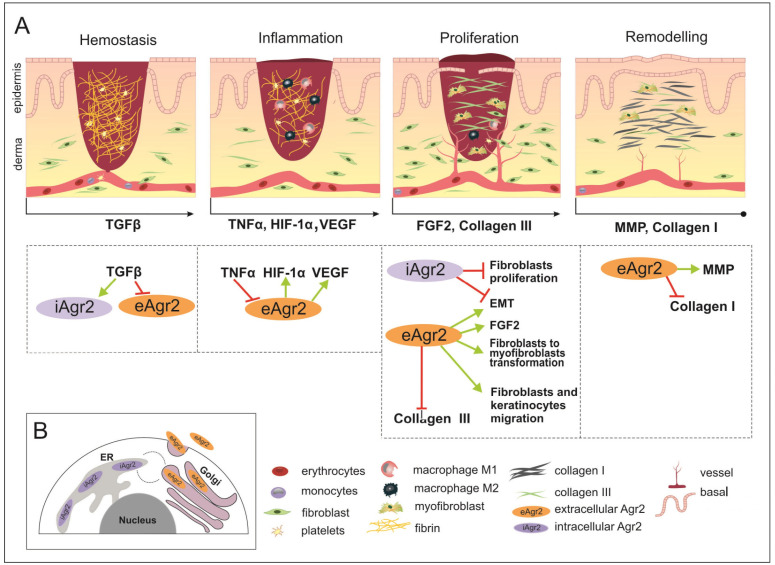
The wound healing (WH) process with appropriate key factors, which might be affected by intracellular AGR2 (iAGR2) or extracellular AGR2 (eAGR2). (**A**) A schematic illustration of four overlapping stages of WH. Arrows located below each of the pictures indicate the start of a specific signature of the WH stage, such as the factor action. This is considered to be due to the connection with AGR2 or the ability to be influenced by AGR2 being superimposed on the skin WH. In the hemostasis stage, platelets give rise to a blood clot formation, in which molecules of fibronectin, vitronectin and thrombospondins form the temporal scaffold structure for the migration of leukocytes and the invasion of keratinocytes, fibroblasts and neutrophils. AGR2 expression is induced upon injury. The inflammation phase starts with the release of mediators such as TNF-α, IL-1β and IL-6, which further amplify the proinflammatory response. The eAGR2 involves [see the boxes below the scheme marked by arrows] negative regulation from TNF-α and activation of VEGF, influencing the range of the proinflammatory reactions. Then, platelets, leukocytes, macrophages and other inflammatory cells activate the following phase, in which angiogenesis, epithelization and fibroblast activation take place. During the proliferation stage, after the appearance of granulation tissue, the wound begins to contract. During the remodeling stage, there is granulation tissue and angiogenesis reduction, the ECM is reorganized by extra collagen deposition, the increase in wound tensile strength occurs from the beginning of the wound healing process to the end of closure. eAGR2 influences collagen III and collagen I deposition, positively regulating keratinocyte migration and fibroblast-to-myofibroblast transformation. The role of AGR2 in EMT is dependent on intra- or extracellular protein localization. The increasing ratio of eAGR/iAGR seems to be responsible for a set of different impacts on cancer cell migration, invasiveness and the progress of metastasis. However, the precise mechanism(s) regulating the levels of AGR2 and/or their balance remain to be further investigated. (**B**) The scheme of AGR2 cellular localization. The scheme presents the ER-resident iAGR localization, which is connected with PDI function and the (UPR) signaling pathway. The AGR2 proteins undergoing the secretory route are present in the Golgi complex, from which they can be returned back to the ER due to KTEL-receptor signaling or secreted to the extracellular space as eAGR.

In certain tumor systems, both TGF-β and TNF-α have been shown to inhibit eAGR2 expression (Figure 3A) [56].

It is important to note that fetal serum has significantly lower levels of PDGF-AB and TGF-β1 compared with adult serum [53]. However, TGF-β is not present in the fetal dermis, but upon wounding, the expression of iAGR2 is activated in fetal post-wound skin of mice (Figure 2) [24,50]. However, the relationship between AGR2 and TGF-β may be complex and context dependent, and further research is necessary to fully understand its implications for cancer progression and profibrotic changes.

### 3.2. Inflammation

Blood clot platelets with secretion of both TGF-β and PDGF take part in the massive cell death at the site of injury and produce TSLP, IL-25, IL-33, which in common activate IL-4/ IL-13-producing cells and recruit macrophages to the wound area [51]. Th2 cytokines IL-4/ IL-13 have a pleiotropic effect on macrophages: they promote macrophages to activate wound healing when colocalized along with apoptotic cells and induce macrophages to produce IL-10 with anti-inflammatory activity, and tissue remodeling growth factors such as TGF-β and matrix metalloproteinases (MMPs) [94]. M2 macrophages with reparative function in wounds express the markers Arginase1 and RELMα (*Retnla*), which in turn promote matrix deposition [85]. At the beginning of the inflammation phase, macrophages that have migrated to the wound area are characterized by secretion of the pro-inflammatory mediators, such as IL-1, IL-6, IL-12, TNFα, and iNOS, in contrast to IL-10 at the low level of secretion, which corresponds to the M1-polarized macrophages [84]. The macrophage phenotype switches gradually to an alternatively activated form known as M2, which is characterized by the secretion of TGF-β1 in common with PDGF, while macrophage recruitment to the damaged area continues (Table 1) (Figure 2) [85,86,87]. Macrophages participating in wound healing also produce VEGF, which stimulates multiple components of the angiogenic cascade [69]. In addition to secretory activity, macrophages phagocytize remaining debris [95]. At the end of the inflammatory phase, T-lymphocytes and B-lymphocytes migrate to the wound area, and although the role of B-lymphocytes in this process is not completely clear, it is believed that T-lymphocytes take part in the cross-linking of collagens in the wound matrix [96]. Genetically modified mouse models with deficient TNF-α exhibited accelerated wound closure. The lack of polymorphonuclear leukocytes and enhancement in angiogenesis and collagen content were shown [57]. In this case, negative regulation of eAGR2 by TNF-α could decline wound closure (Figure 3A). Mice experimental models with a lack of key inflammatory factors for skin repair showed a change in the rate of healing, which resulted in increased scarring [58]. Nuclear factor erythroid 2-related factor 2 (Nrf2) knockout mice, with a deficiency of transcription factor that regulates keratinocyte growth factor, manifest positively regulated prolonged inflammatory response and protracted expression of IL-1β and TNF-α (Table 1) [59]. NRF2 specifically activates the HIF-1α promoter in normoxic conditions and maintains constitutive HIF-1-α expression. NRF2 is downregulated in hypoxia and restored following reoxygenation; this feedback regulation was demonstrated by in vitro studies in murine renal/kidney tubular epithelial cells [97]. This complex regulatory network participating in wound healing possibly includes the AGR2 factor, which was specifically upregulated in response to the depletion of oxygen (Figure 3A) [67]. In comparison with wound healing of adult skin tissue, the fetal healing process is characterized by reduced inflammation (Figure 2). Fetal neutrophils express lower levels of adhesion molecules compared to adult neutrophils and display a decreased ability to recruit to the injury site in comparison with such processes in an adult skin injury. The study of human second-trimester fetal skin demonstrated low levels of immune cells and leukocyte chemokines compared to adult skin. The most important deficiency was found in the CD45(+) leukocyte population. The discrepancy between fetal and mature skin was demonstrated in murine models [88]. The overall number of macrophage is lower in fetal wounds than that in adults, with a high ratio of anti-inflammatory (M2) to M1 pro-inflammatory (M1) macrophages recruited in the wound bed in early gestation steps during mouse development (Table 1). In addition, the presentation time of inflammatory cells is reduced compared to an adult wound.

The role of AGR2 in maintaining intestinal homeostasis and pathogenesis of the inflammatory bowel disease was studied on the model of intestinal epithelial cells culture Caco-2 [60]. After cell exposure to TNF-α, the levels of both mRNA and the protein AGR2 decreased, which was accompanied by an increase in epithelial permeability, which was estimated by transepithelial electrical resistance. The overexpression of AGR2 in Caco-2 had a protective role, as the decrease in epithelial barrier hyperpermeability was demonstrated [60]. The increased expression of Tight Junction proteins and stabilization of intracellular cytoskeletal structures was shown in the Caco-2 epithelial cell line with AGR2. Moreover, AGR2 induced inhibition of NF-κB p65 translocation into the nucleus, thus mediating the negative regulator of the NF-κB-dependent axis [37]. It seems that AGR2 has no potential involvement in the first stages of wound healing. However, it is important to mention that both TGF-β and TNF-α are actively secreted during the inflammation stage and have been shown to inhibit AGR2 expression in tumor systems (Table 1). So, it can lead to a lower contribution at the beginning of the proliferation stage.

### 3.3. Proliferation

In the proliferative phase, the wound surface closes, granulation tissue forms, and the vascular network restores. The secretion of TGF-β1, in common with PDGF, is partly responsible for initiating the proliferative phase and the initial migration of fibroblasts and perivascular progenitor cells (also known as pericytes) into the developing granulation tissue matrix. PDGF destabilizes perivascular progenitors that are associated with blood vessels, allowing them to migrate into the wound bed (Table 1) [52].

In vitro experiments on mouse fibroblast cultures revealed that extracellular recombinant AGR2 controls the migration velocity and directional persistence of cells. The mechanism of action is presumably through the FAK pathway (focal adhesion) and the JNK pathway, since their inhibition results in a significant decline in cell migration [24]. 

Growth factors such as VEGF, PDGF, bFGF bind to endothelial cells receptors, thereby activating intracellular signaling cascades. Activated endothelial cells secrete proteolytic enzymes that lyse BM, so endothelial cells can divide and migrate into the wound. During proliferation, endothelial cells release MMPs, which lyse surrounding tissues for further endothelial proliferation [70]. 

The AGR2 protein was revealed to be a chaperone-like enhancer of VEGF and FGF2. AGR2 can directly bind to these molecules and enhance their homodimerization, thus controlling angiogenesis, endothelial cells and fibroblast invasion (Table 1) [45]. In the prostate cancer cell line PC-3, extracellular AGR2 was shown to enhance VEGF receptor activity through the formation of disulfide bonds and activate the NF-κB pathway [72]. 

Tissue hypoxia stimulates angiogenesis through hypoxia-inducible factor 1 alpha (HIF-1α), which induces the expression of VEGF and endothelial cells to migrate towards low-oxygen areas. It has been shown that in the middle of the wound, the partial pressure of oxygen can be lower than 10 mmHg compared to normal tissue levels of 45–50 mmHg [61,62]. Multiple cell types express HIF-1α in response to injury, including keratinocytes, fibroblasts, and infiltrating immune cells. Fibroblast growth and collagen synthesis require hypoxic conditions; for example, collagen synthesis begins at a pO2 of 10 to 20 mmHg, and at 25 mmHg it becomes maximum. Additionally, overexpression of HIF-1α during wound healing recruits MSCs and stimulates regeneration (Table 1) [63].

During neovascularization, hypoxia decreases, which leads to a fall in the lactate level in tissues and a decrease in the new vessel growth, but collagen synthesis continues. Further, endothelial cells express FGF2 under the hypoxia conditions and hypoxia induced the expression of both FGF1 and FGF2 in macrophages [64,65].

FGF family members control cell proliferation, differentiation and migration. FGF1, 2, 5, 7, 10 are upregulated during cutaneous wound healing. Different FGFs are expressed throughout embryogenesis, where they act as morphogens [73]. In the mouse model, it was shown that after wounding, expression of FGF7 and FGF10 was downregulated in fetal scarless wounds (Figure 2). FGF2 expression decreased in both scarless and scarred fetal wounds [74]. On average, in scarless wound healing and in the fetus, FGF signaling was found to be downregulated (Table 1).

Unlike adult skin, HIF-1α expression was detected in intact fetal skin, which means that HIF-1a constitutive presence in skin before injury can be involved in scarless embryonic wound healing [66]. AGR2 induces lactate production and glucose uptake and was shown to induce the expression of several important enzymes such as lactate dehydrogenase A (LDHA), phosphoglycerate kinase 1 (PGK1), kallikrein 2 (HK2), and enolase 1-α (ENO1), which participate in glucose uptake and lactate production. Moreover, AGR2 induces HIF-1α (Table 1) [15]. An investigation of glioblastoma multiforme tumor (GBM) cells showed that AGR2 expression was regulated by HIF-1α and directed glioblastoma cell growth and the vascularity of tumors (Figure 3A) [68].

Re-epithelialization is provided by keratinocytes recruited from the wound edge and the hair follicle stem cells (SCs) (bulge and gland) [80]. The process is activated by signaling pathways triggered by wound edge cells secreting EGF, KGF, IGF-1, and NGF, a variety of different cytokines and growth factors, in response to injury (Figure 2) [81]. Cell migration proceeds due to the weakening of intercellular contacts and the cytoskeleton’s reorganization. After basement membrane damage, the contact inhibition and physical tension of cell contacts decrease, the SRC kinases activate, and the membrane permeability increases, resulting in a rearrangement of the cell tonofilaments and the start of migration [7]. In addition, collagenase and elastase weaken the cell contacts, and as a result, keratinocytes can migrate along the fibrin of the blood clot in the upper layers of granulation tissue along the chemotactic gradient formed by cytokines [82]. Rho GTPases regulate cell migration, during which cytoskeletal actin fibers are polymerized and new focal contacts with the ECM are formed due to the activation of integrins [98]. When GTPases stop working, the cytoskeleton is reorganized, actin fibers in filopodia are degraded, and a new epithelial layer with contacts is formed that closes the wound [7].

In vivo experiments on mice demonstrated that recombinant AGR2 accelerated the migration of epithelial cells to form elongated migrating tongues. Additional confirmation was obtained using mouse cell cultures (fibroblasts and keratinocytes) treated with mitomycin C, which excluded the proliferation influence (Table 1) [24]. AGR2’s ability to control cell migration, e.g., induce accelerated re-epithelialization, looks very promising in terms of regeneration capacity improvement.

The last step in the proliferation phase is the formation of granulation tissue mass. Being a temporary tissue, it replaces the provisional wound matrix, the main components of which are fibrin and fibronectin, and during this process it can form a scar [52,83]. Additionally, the tissue is characterized by a large number of fibroblasts, granulocytes, macrophages, capillaries and poorly organized collagen bundles. In addition, since angiogenesis is not yet fully complete, this tissue is highly vascularized.

As fibroblasts migrate into the wound bed and interact with the components of the newly synthesized wound ECM, they undergo phenotypic changes triggered by TGF-β1, becoming myofibroblasts expressing α-SMA [55]. Myofibroblasts are rapidly migrating cells that contribute to wound margin contraction. Moreover, myofibroblasts secrete collagens and components of the ECM, for example, fibronectin, glycosaminoglycans, proteoglycans and hyaluronic acid, as well as MMPs that regulate matrix stiffness. The ECM provides a scaffold for cell adhesion and regulates cell growth, movement and differentiation through its stiffness, which means it regulates the activity of fibroblasts in the wound healing process. At the end of this phase, the number of mature fibroblasts decreases due to myofibroblast differentiation and apoptosis [75]. Human fetal skin samples being xenotransplanted in nude mice either subcutaneously (a) or cutaneously (b) demonstrated scarfree (a) and scar (b) wound healing [99]. Scars in cutaneous fetal grafts could be the result of the infiltration of the murine granulation tissue with disposing collagen. It was shown that during wound healing, adipocytes are regenerated from myofibroblasts, cells that are considered differentiated and non-adipogenic. The reprogramming of myofibroblasts involves newly formed hair follicles that trigger BMP signaling and activation of the expression of adipocyte transcription factors [100].

When examining the differences between fetal and adult wound healing, it is important to note that multiple factors are at play and can affect various stages of the process. Although myofibroblasts secrete the same TGF-β1 in both cases, its impact on the healing process differs between the two (Figure 2) [101]. Different signaling pathways are activated in early human fetal skin cells in comparison with mature fibroblasts, with a prevalence of short-lived forms or phosphorylation of Smad2/3 and c-Jun N-terminal kinase components of TGF-β1 cascade. This altered response to TGF-β1 stimuli might be partly responsible for transition between non-scarring fetal regeneration and scarring postnatal processes.

TGF-β indirectly mediates AGR2 downregulation through Smad signaling controlled by activated MAPKs, as shown in studies on A549 and PANC-1 cells (Figure 3A) [89]. On the other hand, endoplasmic reticulum stress induction in HT-29 epithelial cells activates intracellular expression of AGR2, as well as TGF-β1 and leads to AGR2 secretion in the supernatant. Moreover, fibroblast-to-myofibroblast differentiation can be stimulated by recombinant AGR2 added to the culture medium (Figure 3A) [54]. It is noteworthy that apparently myofibroblast number increases in recombinant AGR2 presence take place without abnormal homeostasis, according to post-wound healing analysis [24].

Judging by studies conducted on human cancer cell lines, the AGR2 effect on EMT, migration and invasion abilities depends on the form of the protein, intracellular or extracellular (Figure 3B) [89,90]. It was shown that extracellular AGR2 promotes epithelial morphogenesis and tumorigenesis by disrupting cell–cell contact, disrupting basal laminin and activating fibroblast-associated cancer invasion (Table 1) [91,92]. On the other hand, intracellular AGR2 protects the epithelial cellular phenotype by preventing EMT induction (Figure 3A) [93].

### 3.4. Remodeling

In the last phase of wound healing, the number of cells that make up the granulation tissue decreases by apoptosis, and the provisional matrix is reorganized. Thus, a mature wound is characterized by a reduced number of both vessels and cells [102]. During wound formation, the components of the extracellular matrix undergo certain changes. Collagen III, which was produced during the proliferation phase, is replaced at this time by collagen I. Type III collagen fibrils are thinner than type I fibrils and oriented in small parallel bundles (Table 1). It should be noticed that the collagen I fibrils formed after injury are oriented along the epidermal surface, while the intact skin fibrils are arranged in a stronger intertwined network. Subsequently, myofibroblasts induce wound contraction through multiple collagen contacts and help reduce the surface of the scar [78]. Finally, the blood circulation processes of angiogenesis are slowed down. At this stage, the foundation is laid for the formation of a scar on the wound [7].

Fetal skin is characterized by a higher ratio of type III (60%) to type I (30%) collagen than the same ratio in the adult skin (only 10% to 20%) (Figure 2) (Table 1). During embryogenesis, the ratio of collagens is declining, but final remodeling takes place only at the postnatal period [79].

In vitro experiments on human fibroblasts transfected with plasmid encoding recombinant newt AG (nAG). nAG expression inhibits fibroblasts proliferation and significantly suppresses collagen I and III in intact cell cultures and after TGF-β treatment, as shown by the BRDU test of proliferation activity. In addition, nAG expression activates MMP, which leads to an increase in collagen degradation [76]. The results were confirmed in a rabbit ear injury model, where a recombinant nAG protein solution was injected into the wound (Table 1) [77]. Lower levels of collagen I and collagen III and higher levels of MMP1 led to a higher degree of scar maturation in experimental wounds compared to controls (Figure 3A). 

## 4. Conclusions

We propose the application of evolutionarily conserved factors, which have demonstrated activity in the regeneration of anamniotes, as a novel approach for developing scarless WH in mammals.

Despite extensive research, the mechanisms underlying WH remain incompletely understood. Therefore, it is imperative to investigate a range of regenerating animals to identify cell types and molecular pathways that can be targeted for the development of new therapies to treat skin injuries and fibrotic conditions. Studies have demonstrated that AGR proteins, including nAG and mAGR2, can enhance mammalian regeneration by decreasing scar formation in fibrotic conditions and controlling the proliferation and migration of keratinocytes to promote wound closure. Although AGR proteins are not typically expressed in adult mammalian skin, they have the potential to accelerate WH and initiate a scarless process in the mucous epithelium of the digestive tract, similar to anamniotes with strong regenerative abilities. The evidence suggests that AGR2 plays a pivotal role in wound healing by promoting it through its adhesion domain, making it a potential target for therapeutic intervention.

The presence or absence of the KTEL domain gives AGR2 a dual nature, making it an intriguing candidate for further investigation as a potential therapeutic agent in WH. AGR2 can exist in two forms: iAGR2, which is found in the ER or on the plasma membrane and plays a role in receptor processing and regulating ER stress, and eAGR2, which is a secreted protein. Although the expression of eAGR2 in cancer cells has been extensively studied, a comprehensive portrait of its functionality and the pattern of iAGR/eAGR ratio in various cell types is yet to be fully determined. It has been shown that eAGR is able to increase the migration rate of skin cells (fibroblasts and keratinocytes) in vitro and in vivo.

Thus, the specific activity of AGR2 at different stages of WH suggests that this factor may be promising for scarless WH and skin regeneration. However, further exploration of the regulatory functions of AGR2 is necessary in order to fully understand its potential therapeutic applications. Despite the progress made in studying the functions of AGR2, limitations remain in the translation of this knowledge into clinical practice. It is necessary to explore the role of induced AGR2 expression in adult mammals, particularly in light of eAGR2 expression as a marker in different human carcinomas. The possibility of using AGR2 to enhance the regenerative potential of mammals still requires further research to avoid adverse alterations in the cell expression profile.

## Figures and Tables

**Table 1:** The list of factors involved in WH that can interact with AGR2. From right to left: the name of each factor, an overview of its role in wound repair, the important difference in expression in embryonic tissues, and the type of interaction with iAGR and eAGR.

Factors	WH Function	Embryo Important Differencies	Interaction with AGR2
PDGF	monocytes recruitment, stimulation of fibroblasts proliferation and migration, promotion of fibroblast-to-myofibroblast differentiation [49,51,52]	lower levels in serum compared with adult serum [53]	eAGR2 promotes fibroblast-to-myofibroblast differentiation [24,54]
TGF-β	neutrophils, macrophages, and fibroblasts recruitment; stimulation of angiogenesis and fibroplasia; initiation of the proliferative phase; stimulation of fibroblasts and pericytes; promotion of contraction of the wound bed; fibroblast-to-myofibroblast differentiation and ECM remodeling [50,51,52,55]	lower levels in serum compared with adult serum and no in intact skin [24,53]	TGF-β inhibits eAGR2 expression; eAGR2 promotes fibroblast-to-myofibroblast differentiation; endoplasmic reticulum stress activates iAGR2 expression with TGF-β1 and AGR2 secretion; iAGR2 is activated in fetal post-wound skin [24,50,54,56]
TNF-α	Promotion of inflammation, cell proliferation and angiogenesis [51]	no significant differences were found	TNF-α inhibit eAGR2 expression in combination with epithelial permeability [56,57,58,59,60]
HIF-1α	Stimulation of angiogenesis, VEGF expression; control of endothelial cells migration, fibroblast growth and collagen synthesis; recruitment of MSCs [61,62,63,64,65]	constitutive presence in intact skin [66]	eAGR2 induces lactate production, glucose uptake and HIF-1α expression; eAGR2 expression is upregulated by HIF-1α [15,67,68]
VEGF	Stimulation of angiogenesis, activation of endothelial cells, proteolytic enzymes secretion, releasing of MMPs for further proliferation and migration [69,70]	lower levels [71]	eAGR2 enhances VEGF homodimerization, thus controlling angiogenesis, endothelial cells and fibroblasts invasion [45,72]
FGFs	control cell proliferation, differentiation and migration; FGF 1, 2, 5, 7, 10 upregulation during cutaneous WH; in scarless WH FGF signaling downregulates; FGF2 expression increases under the hypoxia [51,64,65,73,74]	constitutive presence in intact skin as morphogens; FGF7 and FGF10 downregulate in fetal scarless wounds; FGF2 expression decrease in both scarless and scarring fetal wounds [73,74]	eAGR2 enhances FGF2 homodimerization, thus controlling angiogenesis, endothelial cells and fibroblasts invasion [45]
MMPs	Regulation of matrix stiffness, lysing surrounding tissues, control of ECM remodeling [70,75]	no significant differences were found	nAG activates MMP, increase collagen degradation [76,77]
Collagen I, III	collagen III (proliferation phase)-thin fibrils, part of the provisional matrix replaced by the collagen I, orientating along the epidermal surface; low ratio of type III (10%) to type I (20%) collagen [78,79]	higher ratio of collagen III (60%) to collagen I (30%) [79]	nAG suppresses collagen I and III synthesis, increases collagen degradation [76,77]
Cell capabilities	keratinocytes-proliferation, migration along the fibrin of the blood clot, closure of the wound surface [7,80,81,82]; fibroblasts-recruitment to the wound bed, transformation to myofibroblast, secretion ECM [52,55,75,83]; M1-macrophages migration, secrete pro-inflammatory mediators, switch to M2 form [84]; M2-macrophages secrete TGF-β1, PDGF [85,86,87]	lower number of M1, M2-macrophages, higher ratio of M2-macrophages to M1, reduced presentation time [88]	eAGR2 accelerates fibroblasts and keratinocytes migration; eAGR2 promotes epithelial morphogenesis, disrupts cell–cell contact and basal laminin; iAGR2 prevents EMT induction [24,89,90,91,92,93]

## Data Availability

Not applicable.
